# Supplementary figures and images for: The endoplasmic reticulum membrane protein complex localizes to the mitochondrial - endoplasmic reticulum interface and its subunits modulate phospholipid biosynthesis in Trypanosoma brucei
Source: PLoS Pathog. 2022 May 2;18(5):e1009717. doi: 10.1371/journal.ppat.1009717 (PMC9113592; doi:10.1371/journal.ppat.1009717)

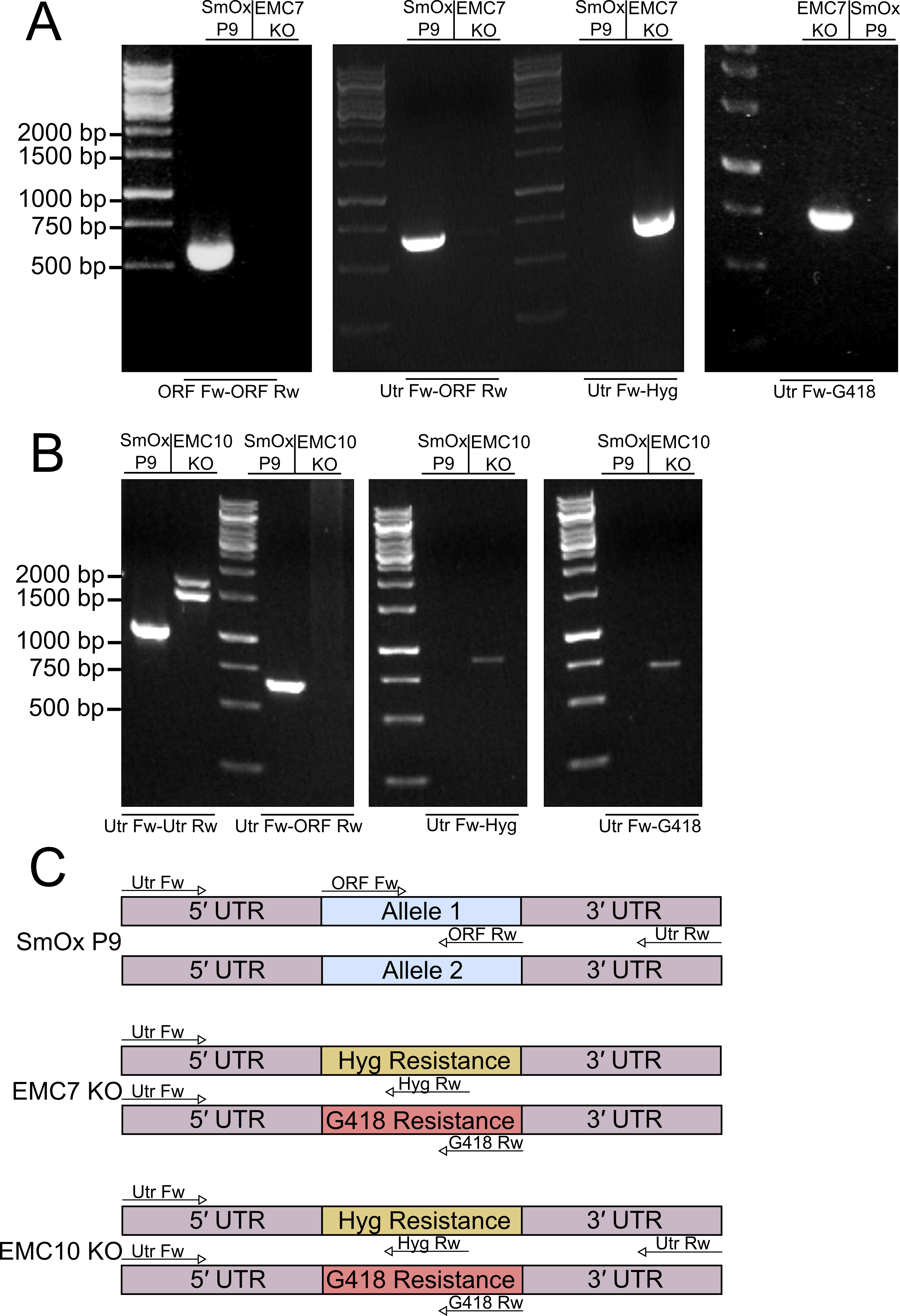

Supplement: S1 Fig — gDNA extracted from parental (SmOx P9), TbEMC7-KO (A) and TbEMC10-KO (B) parasites was analyzed by PCR using the primer pairs indicated in C. The TbEMC alleles and hygromycin (Hyg) and geneticin (G418) resistance cassettes are indicated. (TIF) [file ppat.1009717.s001.tif]

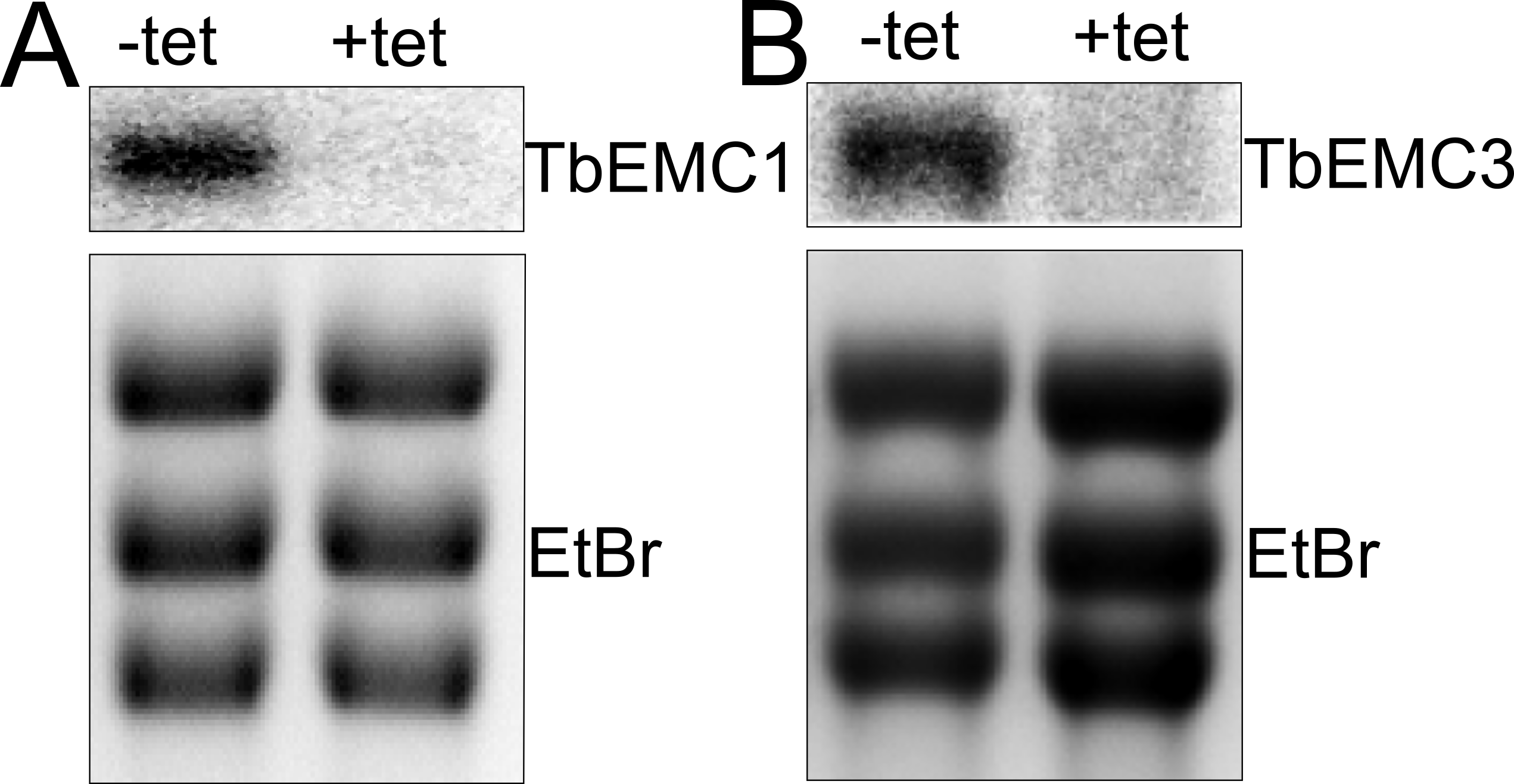

Supplement: S2 Fig — Total RNA extracted from parasites before (-tet) and after 3 days of induction of RNAi (+tet) was analyzed by Northern blotting using 32P-labeled probes against the ORFs of TbEMC1 (A) or TbEMC3 (B). Ethidium bromide-stained cytosolic rRNA served as loading control. (TIF) [file ppat.1009717.s002.tif]

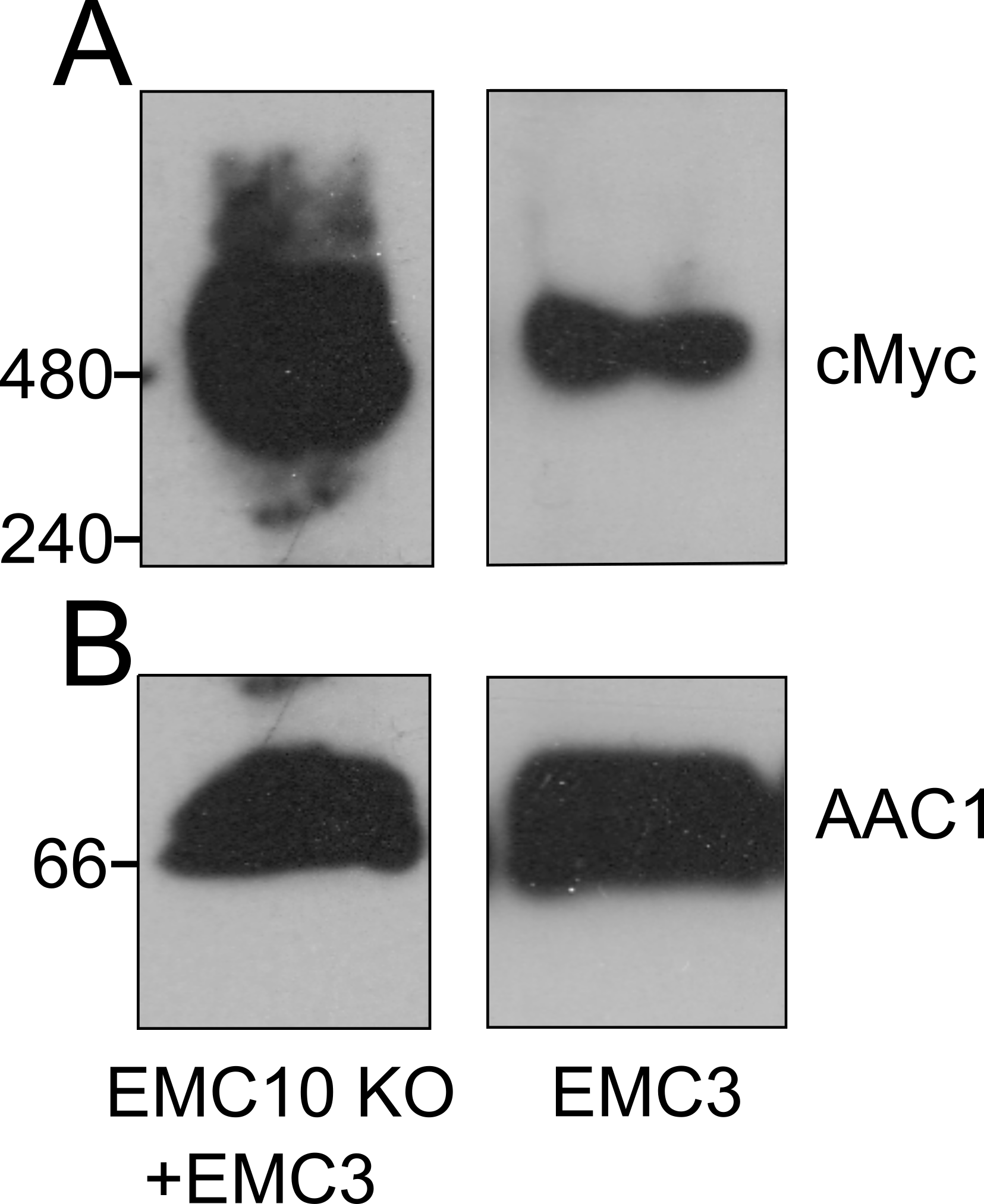

Supplement: S3 Fig — Protein extracts from TbEMC10-KO (left lanes) and control (right lanes) parasites expressing in situ cMyc-tagged TbEMC3 were analyzed by native PAGE and immunoblotting using anti-cMyc (A) and anti-AAC1 (B, loading controls) antibodies. AAC1, ADP/ATP-carrier protein 1. Molecular mass markers (in kDa) are indicated in the left margin. (TIFF) [file ppat.1009717.s003.tiff]

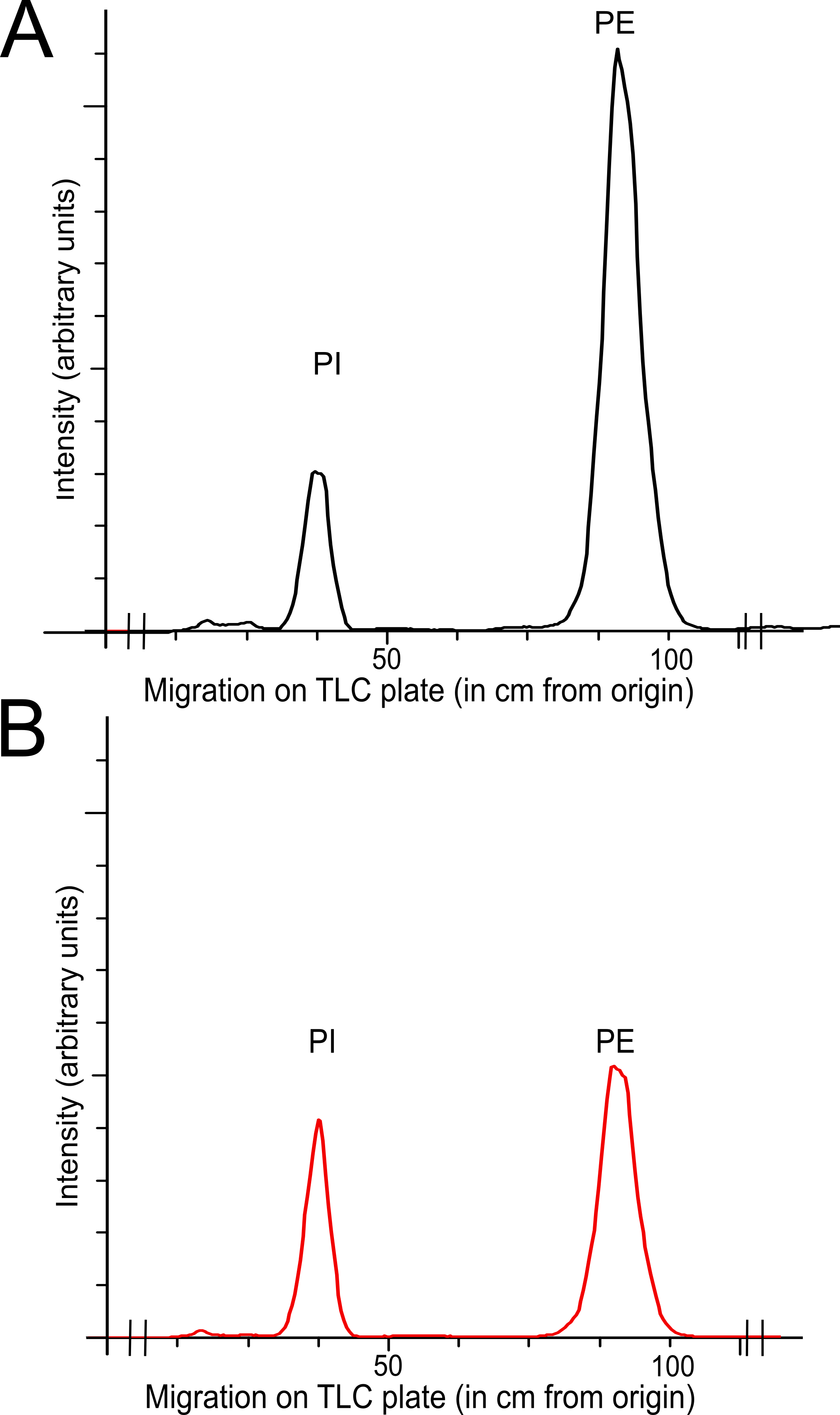

Supplement: S4 Fig — Parasites were cultured in the absence (A) or the presence (B) of tetracycline to maintain or ablate, respectively, TbEMC8 expression and incubated with [3H]-ethanolamine and [3H]-inositol (as control) for 4 h. Phospholipids were extracted and separated by TLC and incorporation of radioactivity into the different phospholipid classes was analyzed by radioisotope scanning. (TIFF) [file ppat.1009717.s004.tiff]

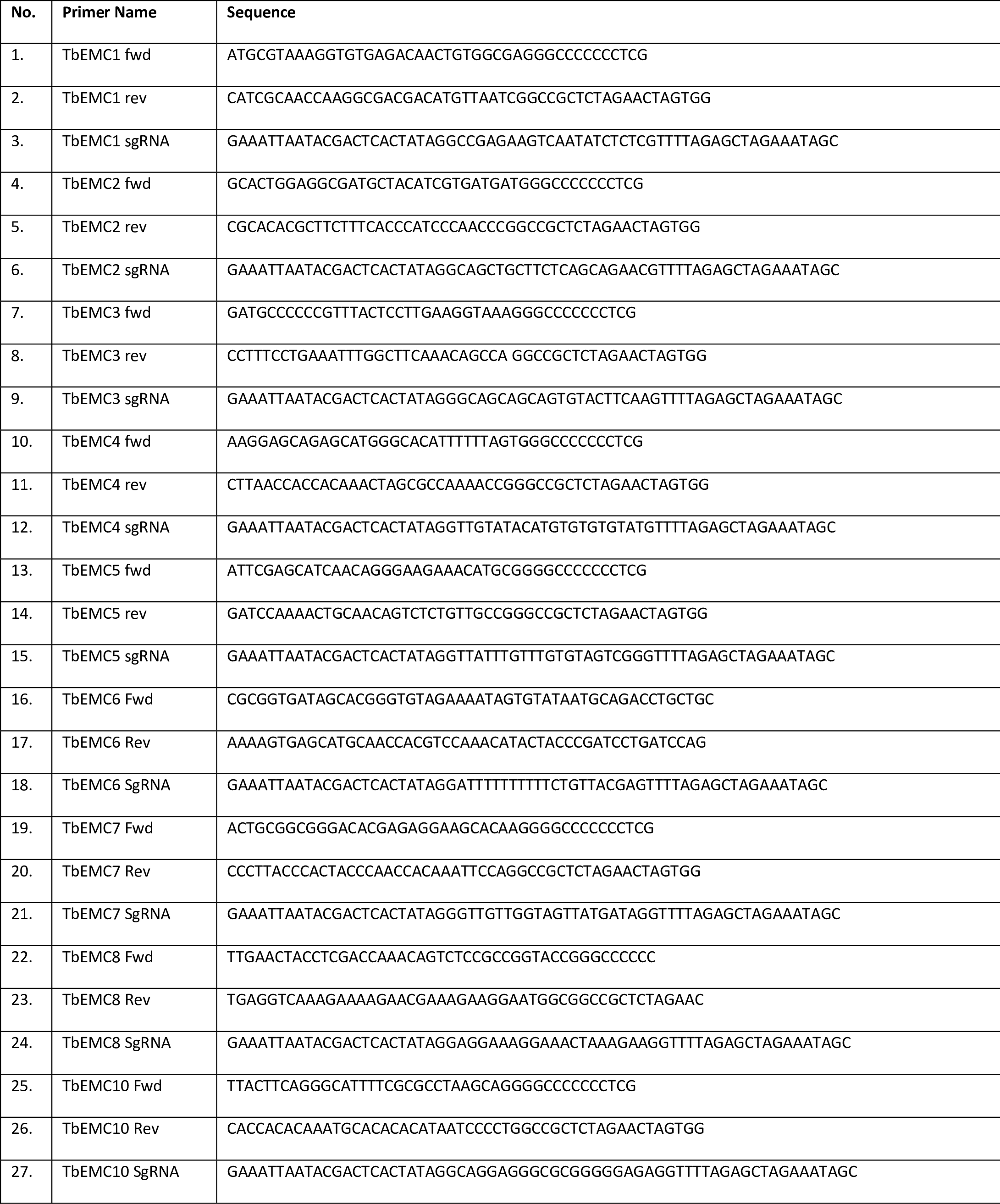

Supplement: S1 Table — (TIF) [file ppat.1009717.s005.tif]

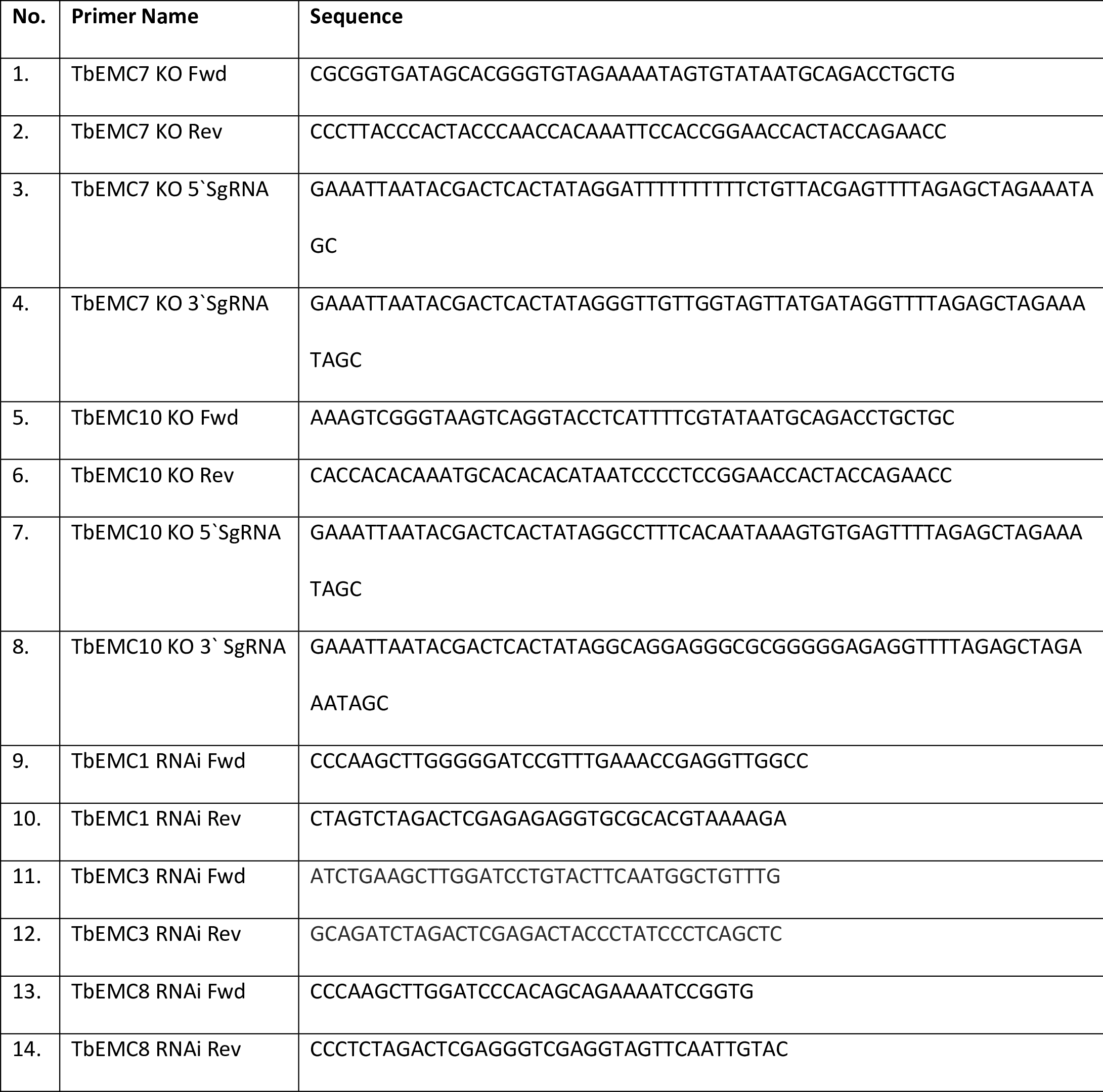

Supplement: S2 Table — (TIF) [file ppat.1009717.s006.tif]

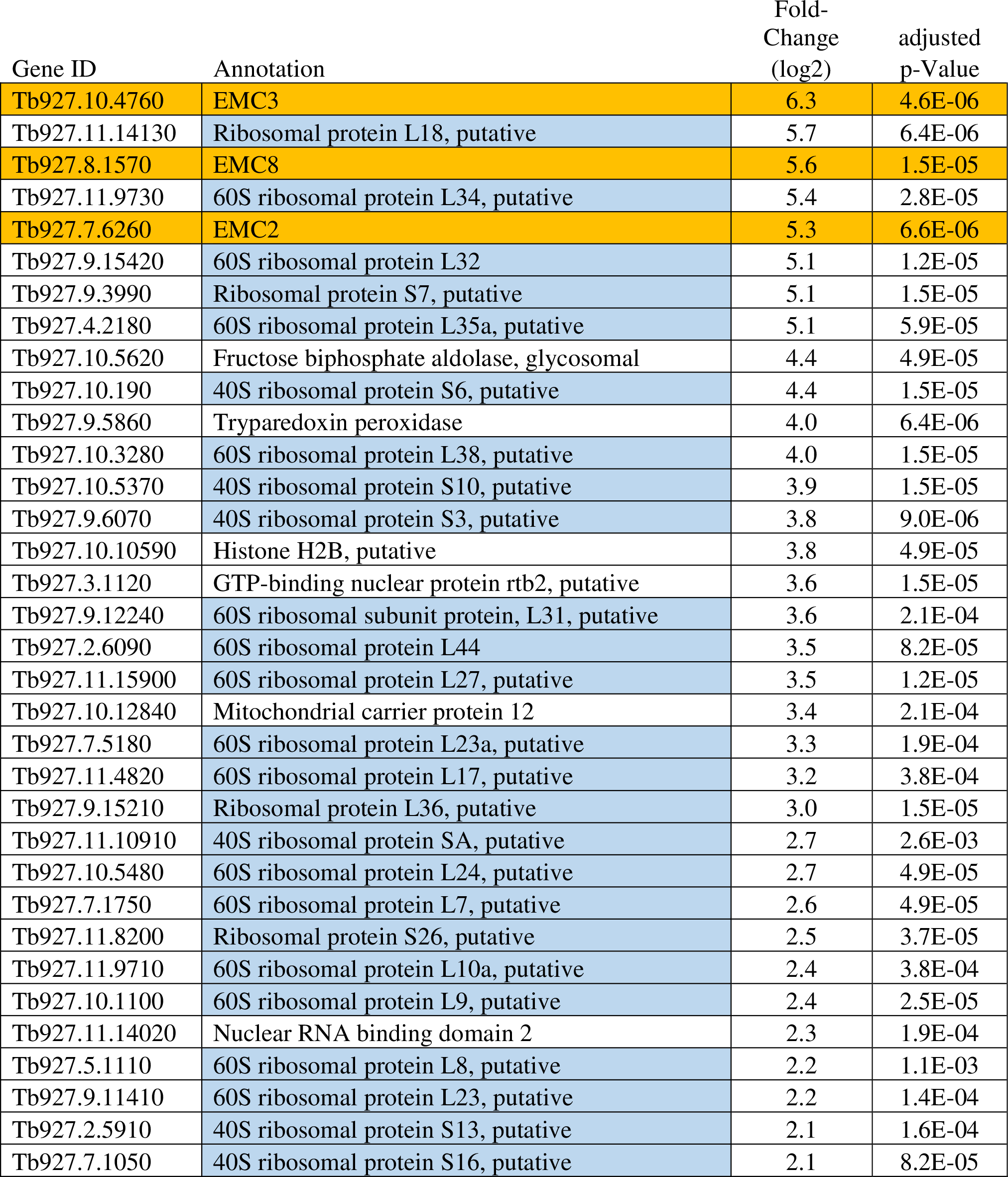

Supplement: S3 Table — (TIF) [file ppat.1009717.s007.tif]

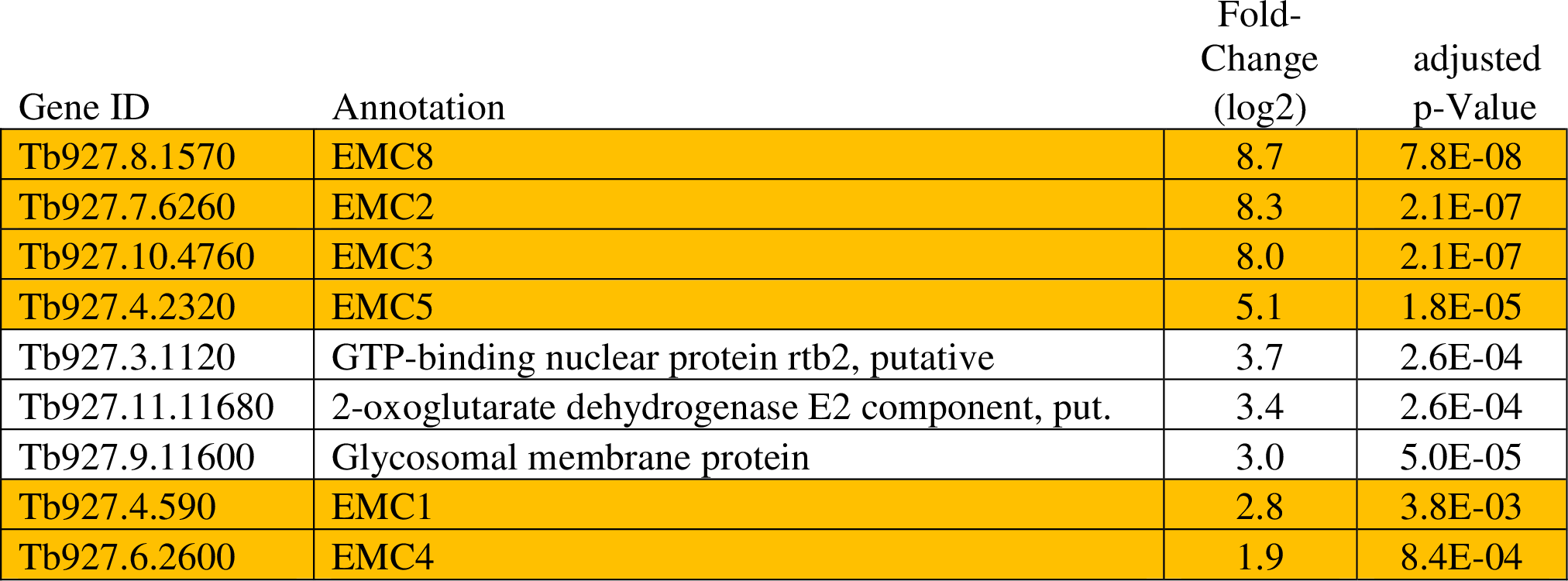

Supplement: S4 Table — (TIF) [file ppat.1009717.s008.tif]
